# Unicompartmental Knee Arthroplasty Provides Superior Clinical and Radiological Outcomes Compared to High Tibial Osteotomy at a Follow-Up of 5–8 Years

**DOI:** 10.3390/jcm12165387

**Published:** 2023-08-19

**Authors:** Markus Neubauer, Eva-Maria Reinberger, Dietmar Dammerer, Lukas B. Moser, Johannes Neugebauer, Florian Gottsauner-Wolf, Stefan Nehrer

**Affiliations:** 1 Center for Regenerative Medicine and Orthopaedics, University for Continuing Education Krems, Dr. Karl-Dorrek-Str. 30, 3500 Krems, Austria; markus.neubauer@krems.lknoe.at (M.N.); dietmar.dammerer@krems.lknoe.at (D.D.); lukasbenedikt.moser@krems.lknoe.at (L.B.M.); 2 Department of Orthopaedics & Traumatology, Karl Landsteiner University of Health Sciences, University Hospital Krems, Mitterweg 10, 3500 Krems, Austria; eva-maria.reinberger@krems.lknoe.at (E.-M.R.); johannes.neugebauer@krems.lknoe.at (J.N.); gottsauner@me.com (F.G.-W.)

**Keywords:** high-tibial osteotomy, unicompartmental knee arthroplasty, joint preservation

## Abstract

Background: Knee Osteoarthritis (OA) is a debilitating disease. Initially, the medial compartments are affected in most cases. For this pathology, joint preservation is preferable. Two surgical procedures aim to meet this goal: high-tibial osteotomy (HTO) and unicompartmental knee arthroplasty (UKA). The aim was to compare clinical and radiological outcomes of HTO versus UKA in patients with unicompartmental, medial OA. Method: Retrospective case series. A total of 86 (61 UKA, 25 HTO) patients that received either treatment at a single, specialized center were assessed pre-operatively and at a single follow-up examination at 77.13 months (±8.170). The Knee Society Score (KSS), range of motion (ROM), SF36 questionnaire and the Tegner score were used. The Kellgren–Lawrence score was assessed pre- and post-surgically. Survivorship with the endpoint “revision” was assessed. Results: The UKA group showed significantly better improvements in KSS scores for pain (*p* < 0.006) and function (*p* < 0.001). OA progression (*p* < 0.02) and survivorship (*p* < 0.018) differed, significantly favoring UKA. ROM, SF36 and Tegner score did not differ significantly. Conclusions: The presented mid-to long-term data suggest that UKA provides superior results in selected outcomes. Nevertheless, significant differences in the demographics of treatments indicate the challenge of comparing these two treatments.

## 1. Introduction

Osteoarthritis (OA) is a common, debilitating disease characterized by joint degeneration. It is thus associated with an increasing loss of joint function, leading to a decrease in the affected patients’ mobility. One-third of OA patients have only one out of the three knee compartments affected [1]. In around 50% of these patients, the medial knee compartment is predominately affected in contrast to the patellafemoral and the lateral compartment [2,3]. OA is often regarded as a “multifactorial” disease that involves genetic, mechanical and biological factors, among many others. The triad of the three factors of age—inflammation—degeneration seems to play a pivotal role [4]. The risk for disease onset increases with every decade, especially after the age of 45 [5]. Interestingly, weight bearing and non-weight-bearing joints are affected. Cyclic loading during activities such as walking is essential both for the development of cartilage and for the maintenance of it. These mechanisms also cause the cartilage to thicken in the areas exposed to greater load, such as in the anterior-to-posterior and medio-to-lateral zones of the joint [6]. If physiological gait mechanisms are altered—due to trauma, weight gain or any other reasons—this may shift these zones of maximal exposure to loads to regions that are less adapted to it [6]. Joint loading onto diseased or damaged cartilage contributes to its accelerated degeneration [6,7]. Unphysiological loading of non-adapted regions causes cartilage fibrillation and thus a breakdown of the physiological cartilage formation. In turn, OA itself shifts joint loading to unadopted areas, causing a vicious cycle.

This painful condition can be surgically addressed by either unicompartmental knee-arthroplasty (UKA) or by high tibial osteotomy (HTO). Both interventions allow for compartment-specific treatment of the knee joint that preserves bone and soft tissue in contrast to total knee replacements [8]. These two treatment options were shown to be effective and are preferably used in younger and active patients [9,10,11]. A recent Consensus Statement also recommended HTO in younger patients (>55 years) with a lower BMI (<33 kg/m^2^), high demand and also a preference for joint preservation [8].

However, the precise indication for either one in an individual patient remains a subject of debate in the orthopedic scientific community.

“Knee—Joint—Protection” aims to preserve the knee with the least possible alterations of its physiological biomechanics and biology. Knee joint degeneration happens in a step-wise fashion. Anterio-medial OA is the most common manifestation with an intact patellofemoral and lateral compartment [12]. However, TKR is only indicated for end-stage OA [13]. Thus, treatment of degenerative knee diseases should be performed in line with the pathological stage. Interestingly, both procedures—UKA and HTO—emerged in the 1960s. A historic perspective may give a better understanding of the rationales for both procedures.

The concept of UKA and total knee arthroplasty or total knee replacement (TKR) were introduced in the 1960s. Initially, UKA showed higher revision rates and less clinical efficacy compared to TKR [14]. When the UKA was first introduced, outcomes were inferior to TKR. Kozin and Scott developed strict inclusion criteria such as age < 60; weight < 82 kg and a range of motion > 90°, amongst others [15]. Potential contributing factors for the poor outcomes were immature designs, narrow indications and more demanding surgical skills [16]. However, this situation has changed dramatically over the course of the past decades, with standardized surgical techniques and designs showing advantages in comparison to TKR for many affected patients [17].

In comparison to the HTO procedure, the remaining concerns regarding UKA include loosening of the implant, or that HTO provides superior functional outcomes for younger and active patients [18,19].

The HTO procedure was first introduced by Jackson and Waugh in the 1960s [20]. Coventry further popularized the surgical treatment predominantly for medial compartment OA in varus-malaligned knees [21]. The rationale was to reduce pain by off-loading the weight from the medial compartment more to the lateral one and to postpone or even avoid the necessity of TKR by stopping or decelerating joint degeneration. Proper patient selection and exact pre-operative axis analysis are pivotal to achieve the favorable results that were shown for this procedure. The most common indication for HTO is a primary or secondary isolated, medial compartment OA with a varus deformity [22]. Factors that are associated with a poor outcome are severe joint destruction like in the case of Ahlback’s disease, high age of 65+, additional patellafemoral OA, a poor range of motion below 90° and joint instability [22]. Another, long-known risk factor for non-union is heavy smoking [23]. The issue of obesity is controversially discussed. However, many authors agree that in the case of obesity, HTO is likely more appropriate than UKA [24].

**Figure 1:**
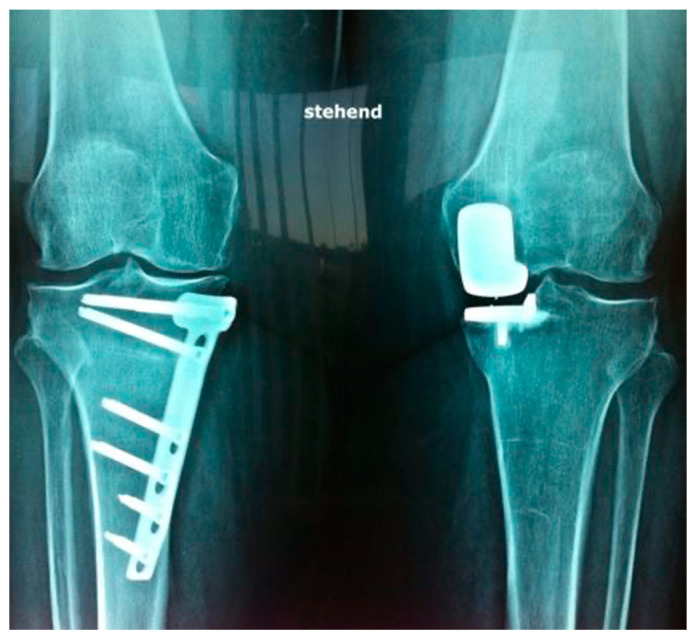
Post-surgical X-ray a.p. (standing) from patient after HTO (right knee) and UKA (left knee).

Also, the influence of prior HTO on later UKA outcomes is still a matter of debate [22]. Moreover, recently published data indicate potentially superior results of the UKA approach regarding durability and post-surgical activity levels in comparison to HTO [25]. However, in terms of survival, HTO and UKA show comparable results [10,26]. HTO patients did show a higher range of motion, whereas the pain level was lower in UKS patients [10,26]. Nevertheless, a Cochrane review on the topic of treating knee OA showed that there is no overall difference between UKA and HTO regarding pain and/or function [27]. Uncertainty remains for optimal indications, with general recommendations in the literature to individually assess factors such as body-mass index (BMI), age, OA grade and activity levels [10]. Nevertheless, a broad consensus in the orthopedic scientific community states that an extraarticular axis malalignment should rather be treated with HTO, whereas an intra-articular malalignment should be addressed with UKA [10,26,28]. Figure 1 illustrates a standing anteroposterior radiograph of a patient after HTO on the right and UKA on the left knee. This study assessed pre- and post-surgical functional scores, pain scores, activity-of-daily-living scores (ADL) and OA progression via radiological scoring at a single follow-up examination. The aim of this study was to elucidate points of uncertainty by comparing the clinical and radiological outcomes of patients suffering from painful, unicompartmental, medial OA who either were treated with HTO or UKA.

## 2. Materials and Methods

### 2.1. Study Design and Patients

This was a retrospective case series. Eighty-six patients between 40 and 65 (UKA: 57.41 ± 0.7267a; HTO: 52.017 ± 1.1972) years of age and a BMI of 30.23 ± 0.705 (UKA group) and 28.84 ± 1.008 (HTO) were included at a mean follow up at 6.43 years (equals 77.13 (±8.170) months). The surgical database of a single, specialized center was used, including patients who had either received HTO or UKA. All included patients met the inclusion criteria. Informed consent was obtained from each participant. This study was approved by the local Ethics Committee (GS4-EK-4/477-2017).

The inclusion criteria were (i) age 40–65, (ii) unicompartmental, medial OA, (iii) signed informed consent form and (iv) surgical treatment performed with either HTO or UKA.

The exclusion criteria included (i) acute inflammatory arthropathy and (ii) additional major surgical and/or medial treatments that may alter activity levels. For the UKA group, post-operative radiographs were evaluated for signs of loosening such as radiolucent lines that may increase in size progressively. The majority of the surgeries were performed by two senior surgeons, including two senior authors (SN, FG), as well as one junior surgeon, including an author (ER). HTO—medial open-wedge osteotomy—was in each case performed by using a plate (TomoFix, by Synthes) and screws. The surgical technique is standardized at this specialized center. Likewise, the UKA was also performed in a strictly standardized manner with cemented, metal-backed, fixed-bearing components (Sigma-Partial Knee, DePuy). Post-surgical rehabilitation protocols included full-weight bearing with crutch support for two weeks in the UKA group and limited weight bearing (2 weeks touch down, 2 weeks 15 kg bearing) in the HTO group. Continuous passive motion (CPM) was used in both groups starting the day after surgery without limitations as tolerated. After 10–12 weeks post-surgery—after a radiological checkup for bone union, implant loosening, etc.—progressive activity was allowed in each group. The clinical outcomes were evaluated by using the Knee Society Score (KSS) with subscales for pain and function, the short-form 36 (SF36) for activities of daily living (ADL), the Tegner score and range of motion (ROM) assessment. The Tegner score has been shown to be a reliable method for evaluating activity levels of patients suffering from knee OA [29]. SF36 was only assessed at follow-up, whereas all other clinical scores were assessed both at baseline and at follow-up. The OA progression was determined by assessing the KL score of the lateral compartment in the UKA group and the total knee joint in the HTO group at follow-up by two independent physicians (ER, MN).

The survival rate was assessed with the endpoint “revision” at follow up. The revision procedure was assessed too. Demographic variables were assessed as potential confounders.

### 2.2. Hypotheses

**H0**: There is no difference between the HTO versus UKA groups concerning clinical scores at follow up.

**H0′**: There is no difference between the HTO versus UKA groups concerning radiological scores/OA progression or revision rates at follow up.

### 2.3. Statistics

Statistical analysis was performed using SPSS software version 22.0 (IBM, Armonk, NY, USA). Data are presented as mean ± standard error of the mean (SEM). An expert for statistics in the life sciences associated with the Karl Landsteiner University for Health Sciences was consulted. A double-tailed, unpaired *t*-test was performed to assess statistical significance for clinical outcome scores as well as patients’ age and BMI at baseline. A chi-square test was performed to assess statistical significance for radiological and revision outcomes as well as patients’ sex at baseline. Assumptions were tested. *p* < 0.05 was considered significant.

## 3. Results

### 3.1. Patients

The two study groups included 86 patients in total with OA of the medial compartment. Of these, 61 received UKA treatment versus 25 HTO cases. The mean age differed between groups and was 57.4 years (SEM ± 0.72) in the UKA group versus 52.017 (SEM ± 1.19) in the HTO group. Also, sex distribution between groups differed, with a male:female ratio of 1.05:2 in the UKA group versus 1.9:3 in the HTO group. The difference between the mean preoperative KSS scores for all 61 UKA patients and 25 HTO patients was significant (*p* < 0.01). However, BMI did not differ between groups and was 30.23 (SEM ± 0.70; UKA) versus 28.84 (SEM ± 1.00; HTO). These results are displayed in Table 1.

### 3.2. Clinical Scores

The improvements derived from the comparisons of means from the pre–post surgery intra-group differences with the KSS score and its sub-scores of (joint) pain (34.6271 ± 2.5539 in the UKA group versus 20.8750 ± 4.3181 in the HTO group) and function (28.33 ± 2.0913 in UKA group versus 14.5833 ± 3.4567 in the HTO group) showed significant differences (*p* < 0.001 and *p* < 0.006, respectively). These differences were, in both cases, in favor of UKA.

The comparison of post-surgical flexion measured via ROM revealed no significant differences between groups (120.16° ± 1.248° in the UKA group versus 125.0° ± 2.102° in the HTO group).

Also, post-surgical Tegner scores were not shown to differ significantly (4.16 ± 0.139 in the UKA group versus 4.76 ± 0.296 in the HTO group).

The “Quality of Life” assessment using the SF36 questionnaire revealed no significant differences between groups neither for physical (KSK: 45.67 ± 1.11 in the UKA group versus 46.98 ± 1.87 in the HTO group) nor psychical sub-scores (PSK: 48.29 ± 1.47 in the UKA group versus 52.58 ± 2.01 in the HTO group).

All clinical outcome score results are displayed in Table 2.

**Figure 2:**
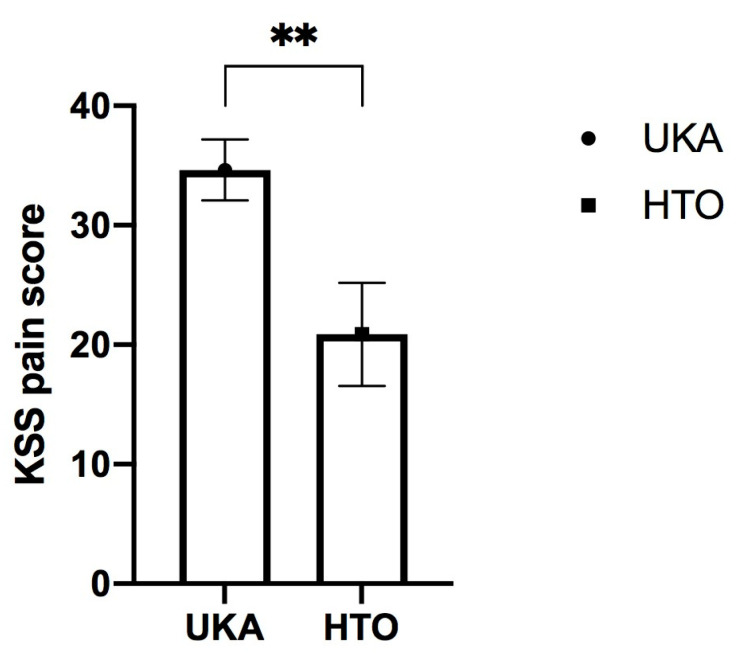
Post-surgical differences of the KSS subscore “pain” at follow up between medial unicondylar arthroplasty (UKA) and high tibial osteotomy (HTO). Results are displayed as Mean ± SEM (** represents *p* < 0.01).

**Figure 3:**
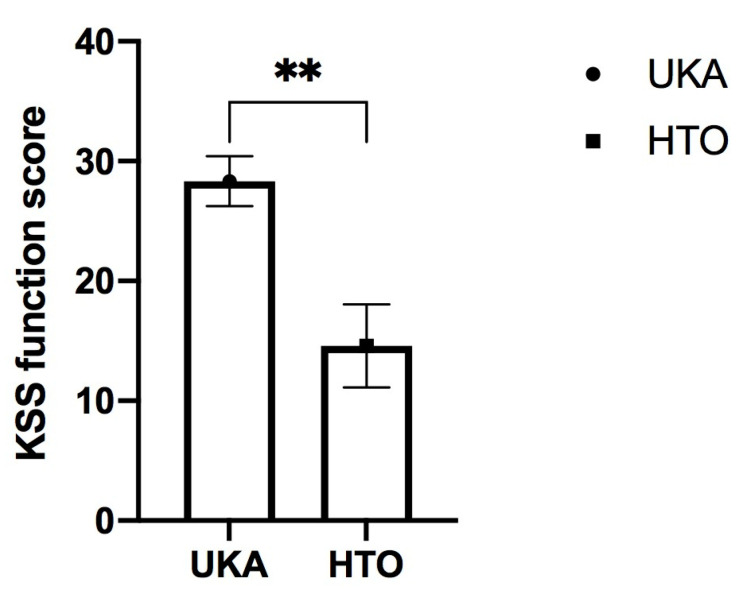
Post-surgical differences of the KSS subscore “function” at follow up between medial unicondylar arthroplasty (UKA) and high tibial osteotomy (HTO). Results are displayed as Mean ± SEM (** represents *p* < 0.01).

The two significant clinical findings are also displayed in boxplot figures (Figure 2 and Figure 3).

With regard to these findings, H0 was rejected for the KSS clinical score.

### 3.3. Radiological Outcome/OA Progression

OA progression, radiologically measured via the KL score, differed significantly, favoring the UKA group (*p* < 0.02) (Table 3).

With regard to these findings, H0′ was rejected for radiological score/OA progression.

### 3.4. Survivorship/Revision

The survivorship with the endpoint “revision” differed significantly between groups, favoring UKA (*p* < 0.018). The revision methods used in the UKA group were one polyethylene inlay change and three uses of TKR. In the case of the inlay change, destabilized and slightly corroded polyethylene was the reason for the revision. The patient reported a mild traumatic incidence before the onset of the knee pain which was the likely reason for presenting to our department.

With regard to these findings, H0′ was rejected for the revision rate. The revision methods used in the HTO group was one UKA and five TKRs. These results are displayed in Table 4.

## 4. Discussion

The potential superiority of either HTO or UKA, when addressing the debilitating condition of medial, unicompartmental knee OA, is still a matter of discussion in the scientific orthopedic community. The aim of this study was to elucidate points of uncertainty by comparing clinical and radiological outcomes of patients after receiving either HTO or UKA. The presented mid-to long-term data suggest that the UKA group had superior clinical outcomes regarding function (*p* = 0.001) and pain (*p* = 0.006) measured with KSS score, a lower OA progression (*p* = 0.02) and a significantly lower revision rate (*p* = 0.018) at follow up. These findings are mostly in line with the recent literature [10,30]. However, a superior range of motion in HTO was not shown [10].

The presented mid-to long-term data further suggest that survivorship was better in the UKA group, with the most common revision being TKR. These findings are also in line with the recent literature [25,31,32]. Despite these differences, other tools assessing function, such as the forgotten joint score—a widely used tool to assess patient-reported outcomes with a focus on joints—did not show differences at a one-year follow-up [33].

UKA does, unlike HTO, resurface the knee and thereby restore a pre-arthritic state. Regarding the revision cases in the HTO group, only one case was converted to an UKA and five cases to a TKR. The recent literature shows that UKA after failed HTO is safe, effective and leads to clinical and functional outcomes that are comparable to TKR [34]. In context of the current literature, some noteworthy points may be discussed. Besides endpoints such as function and pain, other factors should also be considered for the patients’ and surgeons’ joint choice of the surgical procedure.

For example, in a recent systematic review and metanalysis from Shen et al., the revision rate of UKA with revision TKR was shown to be greater in comparison to HTO [35].

Likewise, Serbin et al. described in a recent large, matched cohort analysis that HTO may be converted to TKR later than UKA [36]. Some surgeons may be concerned that the altered anatomy may lead to inferior survivorship results for TKR after HTO. However, the recent literature shows an excellent long-term 10-year survivorship for TKR after HTO [37]. The influence of the surgical procedure upon the progression of the remaining compartments of the joint is another important factor. UKA was more likely to show ligamentous tightness and kinematic abnormalities compared to HTO, which contributes to the progression of early OA in the adjacent knee compartments [38]. Another factor that may concern health-care providers is cost-effectiveness. Recently, Smith et al. showed that HTO is a cost-effective procedure in patients below 60 years of age and UKA was cost-effective in patients above 60 years of age [39]. The same authors concluded that patients’ preferences, as well as their functional utilities, are important factors to consider for the procedure choice.

Body mass index is believed to have an influence upon the failure of UKAs as well as HTOs.

A patient with a BMI greater than 30 is considered to have adiposity. Thus, we want to provide additional information about our study population. In our study subjects, 44% of the UKA group and 20% of the HTO group had a BMI greater than 30. Considering the revisions, the one patient in need of a polyethylene inlay change had a BMI below 30. However, two patients of the three revision cases in the UKA group had a BMI below 30.

In the HTO group, three out of five patients receiving a TKR revision had a BMI below 30. Also, the patient receiving a UKR after HTO had a BMI below 30. These observations indicate a minor influence of BMI over 30 on revision necessity. However, the number of patients was too low to run a sound statistical analysis. These data show that HTO and UKA can be considered to be competitive treatments and that the joint choice of the patient and surgeon should be based on the above-described individual factors. The limitations of this study have to be considered concisely. The majority of the surgeries were performed by two senior surgeons, including two senior authors, following standardized, unaltered procedures (SN, FG). Despite the poorly investigated effect of surgical volume and experience upon the outcome, an effect may be assumed [40]. In this study, the senior UKA surgeon (FG) performs >100 UKAs annually at two major centers. At the single, specialized center, all 25 HTOs and 61 UKAs performed during the recruitment period were included.

A limiting factor is the low number of included patients. Also, the distribution of 25 HTO cases versus 61 UKA cases is an uneven distribution, leading to hurdles in the analysis. Nevertheless, considering the relatively low use of UKA in the overall joint replacements, all patients at this specialized center that met the inclusion criteria in a three-year time frame prior to this retrospective case series were included. Likewise, HTO is in general an infrequently performed surgery. This explains the uneven patient numbers in the groups. Also, all eligible HTO cases were included during the recruitment period. The data presented may further the use of this, in the authors’ opinion, useful UKA approach that also helps to bridge the gap between too-early OA stages for TKR but too-late stages for mere conservative treatments. Furthering the use of UKA may also help to decrease the high rates of TKR being performed annually [41]. Another limiting factor is that the baseline demographic characteristics and KSS scores of the patients were different, which makes a comparison challenging. BMI was not significantly different, but age and sex were. What we saw was that “young men” were more likely to be in the HTO group versus “older women” in the UKA group. However, this phenomenon is common in similar studies [25]. Notably, UKA indications are sometimes stated to exclude patients below the age of 60 [42,43] and only a few, more recent studies have investigated populations of UKA patients under 60 [44]. Therefore, post-operative pain levels may be reduced in UKA, but it is questionable if this difference is beneficial with regard to the aim of joint preservation. Our data suggest that UKA is also a suitable procedure in selected cases to be performed in patients younger than age 60. As a consequence, this rather new insight into the potential benefits of UKA for a younger population may contrast with traditional knowledge that was drawn from older patients. Therefore, this younger age population group is a rather novel one that needs to be separately studied like we did in this paper. As an outlook for the future, patient selection in OA treatment based on OA subgroups is a rather novel approach aiming to tailor more personalized treatment options. For example, up to six different OA phenotypes have been described [45]. Interestingly, one of these six types is called “mechanical overload” [45]. Intuitively, axis-correcting procedures like HTO could be preferable in this group. Analysis stratifying for these subgroups could be of value. To identify patients, biomarkers—radiological, biochemical, mechanical, etc.—may be suitable for designing improved clinical trials and subsequently lead to optimized treatment regimens for OA patients [46]. Potentially, not every biology is suitable for joint-preservation, whereas others may posses more inherent “joint-resilience”. The authors’ conclusion is that HTO does have important indications in this age group, especially so in combination with cartilage regeneration procedures such as autologous chondrocyte transplantation (ACT), bone marrow aspirate concentrate (BMAC) procedures and others. In these cases, joint preservation would not be achievable without axis correction. Procedures like ACT in combination with HTO may also be applicable in early-onset medial OA cases, which is a potentially large group for this procedure [47].

## 5. Conclusions

UKA showed beneficiary results for pain and function that were superior to HTO in a medial OA patient. These findings may promote a wider use of UKA. HTO is an effective procedure, especially for malaligned knees with the aim of joint preservation. Indications for each procedure seem to differ as each procedure targets distinct pathological conditions.

## Figures and Tables

**Table 1:** Baseline characteristics of patients per group.

		UKA	HTO
*n*		61	25
age	Mean	57.418	52.017
	SEM	±0.7267	±1.1972
	*p*-value (∆ at baseline)	0.000 *	
BMI	mean	30.23	28.84
	SEM	±0.705	±1.008
	*p*-value (∆ at baseline)	0.279	
Sex	m:f	21:40	19:6
	% m:f	34.4%:65.6%	76.0%:24.0%
	*p*-value (∆ at baseline)	0.000 *	

Note: BMI = body mass index; f = female; HTO = high tibial osteotomy; m = male; UKA = unicompartmental knee arthroplasty; * = significant *p*-value, ∆ = difference.

**Table 2:** Clinical outcome scores: differences between groups post-surgery.

		UKA		HTO		*p*-Value	Favors
Category		Mean	SEM	Mean	SEM		
KSS	∆KSS function	28.3333	±2.0913	14.5833	±3.4567	0.001 *	UKA
	∆KSS pain	34.6271	±2.5539	20.8750	±4.3181	0.006 *	UKA
ROM (post OP)	ROM flexion	120.16	±1.248	125.00	±2.102	0.056	n.s.
SF36 (post OP)	KSK	45.674869	±1.1122	46.982712	±1.8764	0.537	n.s.
	PSK	48.298241	±1.4709	52.580000	±2.0149	0.092	n.s.
Tegner		4.16	±0.139	4.76	±0.296	0.077	n.s.

Note: HTO = high tibial osteotomy; KSS = Knee Society Score; n.s. = not significant; UKA = unicompartmental knee arthroplasty; ROM = range of motion; SF36 = short form (36): PSK = psychic sum scale, KSK = physical sum scale; * = significant *p*-value; ∆ = difference.

**Table 3:** Radiological outcomes: differences between groups post-surgery.

Category	UKA		HTO		*p*-Value	Favors
OA Progression (KL-Based)	Yes	No	Yes	No		
*n*	22	37	14	7	0.02 *	UKA
% intra-group	37.3	62.7	66.7	33.3		

Note: HTO = high tibial osteotomy; KL = Kellgren–Lawrence; OA = osteoarthritis; UKA = unicompartmental knee arthroplasty; * = significant *p*-value.

**Table 4:** Survivorship (endpoint: revision).

Category	UKA		HTO		*p*-Value	Favors
Revision	Yes	No	Yes	No		
*n*	4	57	6	18	0.018 *	UKA
% intra-group	6.6	93.4	25.0	75.0		
revision method	3 × TKR, 1 × PE		5 × TKR, 1 × UKA			

Note: HTO = high tibial osteotomy; PE = polyethylene (change); TKR = total knee replacement; UKA = unicompartmental knee arthroplasty; * = significant *p*-value.

## Data Availability

Data available on request due to restrictions, e.g., privacy or ethical. The data presented in this study are available on request from the corresponding author.
